# Intelligent Image-Based Railway Inspection System Using Deep Learning-Based Object Detection and Weber Contrast-Based Image Comparison

**DOI:** 10.3390/s19214738

**Published:** 2019-10-31

**Authors:** Jinbeum Jang, Minwoo Shin, Sohee Lim, Jonggook Park, Joungyeon Kim, Joonki Paik

**Affiliations:** 1 Graduate School of Advanced Imaging Science, Multimedia and Film Chung-Ang University, Seoul 06974, Korea; jinbeum23@gmail.com (J.J.); minwoo03d0@gmail.com (M.S.); eunoia0130@gmail.com (S.L.); 2 2iSYS Co. Ltd., Uiwang 15850, Gyeonggi-do, Korea; pjk@2isys.com (J.P.); virtual@2isys.com (J.K.)

**Keywords:** railway inspection, line scan camera, deep learning, single-shot detector, image comparison, Weber contrast

## Abstract

For sustainable operation and maintenance of urban railway infrastructure, intelligent visual inspection of the railway infrastructure attracts increasing attention to avoid unreliable, manual observation by humans at night, while trains do not operate. Although various automatic approaches were proposed using image processing and computer vision techniques, most of them are focused only on railway tracks. In this paper, we present a novel railway inspection system using facility detection based on deep convolutional neural network and computer vision-based image comparison approach. The proposed system aims to automatically detect wears and cracks by comparing a pair of corresponding image sets acquired at different times. We installed line scan camera on the roof of the train. Unlike an area-based camera, the line scan camera quickly acquires images with a wide field of view. The proposed system consists of three main modules: (i) image reconstruction for registration of facility positions, (ii) facility detection using an improved single shot detector, and (iii) deformed region detection using image processing and computer vision techniques. In experiments, we demonstrate that the proposed system accurately finds facilities and detects their potential defects. For that reason, the proposed system can provide various advantages such as cost reduction for maintenance and accident prevention.

## 1. Introduction

Image processing and computer vision algorithms are applied to inspect defects in railways for safety and maintenance, which is called image-based railway inspection system (IRIS). The IRIS automatically detects surface cracks or defects to prevent accidents from RGB or gray-scale images. In various industry fields, existing inspection systems used simple image processing techniques, such as feature extraction, histogram analysis, and graph cut, to name a few [1,2]. Similar to image classification and analysis fields, deep learning can enable more accurate detection of defects in general inspection applications [3,4]. However, it is difficult to inspect structures and facilities in railway environment because of an enormous inspection region.

To inspect a railway, it is important to automatically detect various structures and facilities, such as screws connecting railway tracks, and sleepers and catenary mast supporting power cable. Tracks are usually damaged by the friction between their surfaces and wheels. Electrification systems, such as overhead power lines, are also periodically inspected since they should stably supply electric power to the vehicle. However, conventional inspection systems have long been dependent on human manual observation, which is very inefficient since human observer becomes easily tired and loses concentration on important objects after ten to fifteen minutes. Furthermore, only a very small time slot is allowed for human inspection since trains usually operate all day long.

To solve those problems, various image processing and computer vision-based automatic inspection approaches were proposed [5]. Min et al. detected defects on a track surface by combining several image processing algorithms [6]. Min’s system first finds tracks by analyzing features in the hue channel, and then detects defects using image enhancement and contour-based surface profiling. Karakose et al. also proposed a track diagnosis method for fault diagnosis using Canny edge detection and morphological processing [7]. Han et al. detected insulator defects for catenary maintenance in high-speed railroad [8]. Zhang et al. detected cracks in the wall of a subway tunnel using morphological processing and simple classification method [9]. This method used a line scan camera for high resolution image acquisition. Attard et al. presented a tunnel maintenance system that compares two images acquired at the same spot [10]. Jang et al. proposed a deformation detection system to inspect overhead rigid lines [11]. Recently, deep learning-based railway inspection systems were proposed. James et al. performed segmentation and classification of railroad tracks using a series of deep neural networks [12] based on U-Net [13] and dense network [14]. Gibert et al. also proposed an object detection method using multi-task learning approach for railroad track inspection [15]. This method consists of material and fastener detection branches, which share the same parameters. The fastener branch has another shared network and two-task network structures.

To inspect various facilities, a railroad inspection car (RIC) was developed [16,17,18]. RICs detect various types of defects, such as damage and wear of the facility, using surface profiling, image analysis, and stability test. Although it is efficient to maintain railroad facilities installed across the whole operating sections, the operation for maintenance is still limited during the transport service time. Moreover, it is difficult to deal with repeatedly installed structures in the railway environment since most RICs inspect them using two-dimensional (2D) images acquired while the car is moving forward at a constant speed.

This paper presents a novel railway facility inspection system for efficient maintenance in urban railroad infrastructure with a special focus on overhead rigid lines in subway tunnels. The proposed system obtains images including facilities using a gray-scale line scan camera that can generate an image with wider field of view (FoV) and higher resolution than a general RGB image sensor [19]. The proposed inspection system finds defects by detecting deformed regions by comparing two images acquired at different times. More specifically, the proposed system first performs coarse registration of two images to align positions of same facilities, and then finds main facilities using deep learning-based object detection. For the object detection task, we used single-shot multibox detector (SSD) [20] with a proper modification to improve both detection accuracy and speed. The proposed system then detects deformed regions based on image processing techniques. After finely registering two facility images, we obtain the remarkable deformation regions using Weber contrast-based image subtraction.

This paper is organized as follows. Section 2 describes the image acquisition process using line scan camera, and we present the proposed inspection system in Section 3. In Section 4, the performance of the proposed system is experimentally demonstrated, and Section 5 concludes the paper.

## 2. Image Acquisition Using Line Scan Camera

In this paper, we inspect railroad structures with facilities installed in the underground tunnel. Railroad vehicles are operated by electronic power supplied from overhead lines to pantograph, which are repeatedly installed along the entire driving route, and supporters fitted to the wall. Images acquired using general 2D area-based camera systems with a narrow FoV cannot include the overall shape since the camera installed on the vehicle is too close to the facilities. Although video frame-based stitching methods can be alternatively used to reconstruct the entire shape of the object, they are not suitable for real-time image stitching in the fast-moving vehicle.

The proposed system acquires railway tunnel images using a line scan camera, which was widely applied to various areas such as remote sensing and manufacturing industry. The camera sensor consists of 1×N pixel array, and then generates a 2D image by collecting them in temporal order of acquisition. When an object moves at a constant speed during a specific period, the camera system acquires the high resolution image without stitching and registration processes.

**Figure 1:**
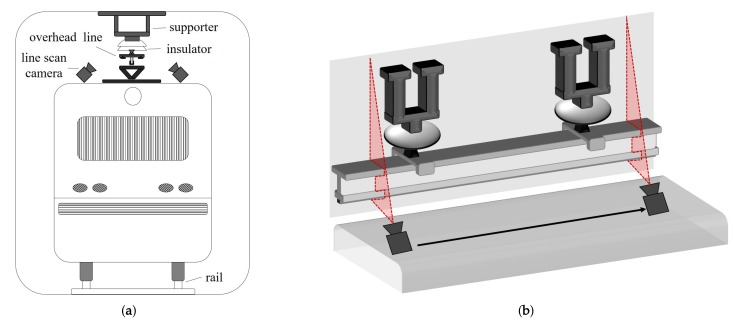
(**a**) Front view of a railroad vehicle in an underground tunnel and (**b**) image acquisition process using a line scan camera.

Figure 1a shows a front view of the vehicle in an underground tunnel. A line scan camera is installed on the top of the railroad vehicle in the orthogonal direction to the vehicle’s moving direction. As shown in Figure 1b, the camera sensor scans narrow horizontal sections, and then stores each scanned result in the form of a one-dimensional (1D) signal. Each line has the resolution of 2×4096 with pixel size of 1.2 mm${}^{2}$. A lighting system is equipped next to the camera system to avoid low light image degradation. Since the train moves at a variable speed, the acquired image is usually distorted when the line scan camera captures at a constant acquisition interval. For that reason, a tachometer is installed on the train wheel to acquire images by controlling frame rate based on the distance traveled for a given time.

**Figure 2:**
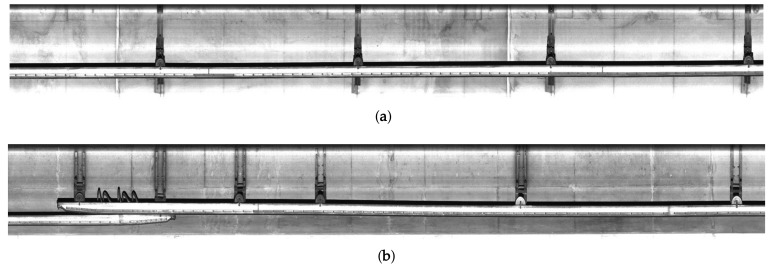
Acquired images: (**a**) the 5-th and (**b**) 20-th frames.

Figure 2 shows acquired images with overhead conductors of subway tunnels using the line scan camera. Each image has the size of 16,834×2048 by rotating $90^{\circ }$ after 2 pixel binning. Since most structures and facilities are within the camera’s depth of field, we can acquire high-quality images despite some degradation problems, such as brightness saturation and noise by airborne dust.

## 3. Facility Inspection Algorithm

### 3.1. Overview

The proposed system automatically inspects structural defects of railway environment. Most facilities installed in the tunnel, such as overhead rigid conductors, are used to supply electric power into the train through its pantograph. Conventional inspection systems detect all candidates of wears and cracks using single image-based processing methods. The conventional image processing-based approach provides an acceptable detection accuracy for a small FoV images. However, it is difficult to distinguish whether detected region is a real defect or not if the image contains complicated frequency components or complex background. Moreover, single image-based systems cannot detect defects caused by structure’s shape deformation and loss of components since most overhead lines and supporters consist of durable metal materials unlike tunnel walls.

**Figure 3:**
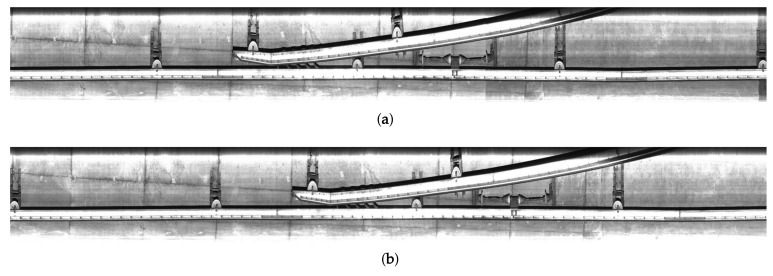
A pair of the images acquired at the same location but at different times. The image (**a**) was acquired before (**b**).

To solve these problems, the proposed system inspects structures and facilities related with overhead lines using a pair of images as shown in Figure 3. The image sets are acquired using the line scan camera at the same spots but at different times. We assume that the reference image set $G^{b}=\left\{g_{0}^{b}\left(x,y\right),g_{1}^{b}\left(x,y\right),\cdots ,g_{n-1}^{b}\left(x,y\right)\right\}$ is acquired before the target image set $G^{a}=\left\{g_{0}^{a}\left(x,y\right),g_{1}^{a}\left(x,y\right),\cdots ,g_{n-1}^{a}\left(x,y\right)\right\}$, and they have no defects such as deformation and loss based on human inspection. The target image set is the one to be inspected. The main objective of the proposed system is to detect deformed regions for maintenance of overhead conductors by comparing two images acquired at different times. We exclude cracks on the tunnel wall as inspect subject in the proposed system since they are simply extracted by single image-based inspection systems.

The proposed system consists of three functional steps: (i) image reconstruction using registration based on phase correlation and image composition, (ii) facility detection using deep learning-based object detection, and (iii) facility inspection using image comparison approach based on Weber contrast. In this section, we describe each step of the proposed system in the following subsections.

### 3.2. Image Reconstruction

Given a pair of reference and target images, the proposed system first reconstructs each image. As shown in Figure 3, positions between corresponding facilities in the same driving section are not initially aligned because of various problems such as different speed and jittering of the camera. In addition, some parts of facilities are often divided into two neighboring frames in the image acquisition process.

**Figure 4:**
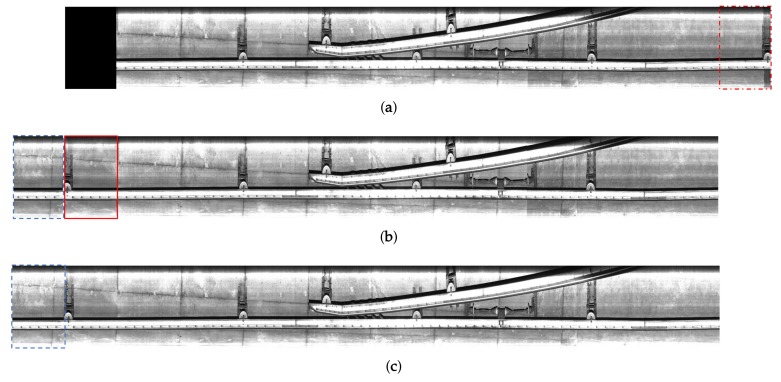
Coarse registration process of the proposed system: (**a**) horizontally transformed image of Figure 3a; (**b**) reconstructed result of (**a**); and (**c**) reconstructed results of Figure 3b, respectively.

The proposed system registers two images using phase correlation. More specifically, disparity or motion vector between two images is estimated by computing correlation in the frequency domain. It is more efficient to coarsely register two large-scale images than spatial domain-based motion estimation methods because of simple multiplication of fast Fourier transformation (FFT). The motion vector (Δx,Δy) obtained by maximizing the phase correlation is defined as

(1) $$\left(\Delta x,\Delta y\right)=\arg\max_{\left(x,y\right)}F^{-1}\left\{\frac{F\left\{g_{i}^{b}\left(x,y\right)\right\}\cdot F^{*}\left\{g_{i}^{a}\left(x,y\right)\right\}}{|F\left\{g_{i}^{b}\left(x,y\right)\right\}\cdot F^{*}\left\{g_{i}^{a}\left(x,y\right)\right\}|}\right\},$$

where $g_{i}^{b}\left(x,y\right)$ and $g_{i}^{a}\left(x,y\right)$ respectively represent the *i*-th frame acquired without temporal synchronization, and F and $F^{-1}$ the Fourier and its inverse transformation operations, respectively. Superscript ‘*’ indicates the conjugate of a complex number and ‘·’ a pixel-by-pixel multiplication. In the proposed method, we translate $g_{i}^{b}\left(x,y\right)$ by the horizontal motion value Δx to prevent deformation of $g_{i}^{a}\left(x,y\right)$ in which we should inspect facilities. The positions of facilities are coarsely aligned by translating $g_{i}^{b}\left(x,y\right)$ using phase correlation as shown in Figure 4a.

Once $g_{i}^{b}\left(x,y\right)$ is translated, we lose the left and right parts of the image. When we obtain the negative motion value, the translated version of $g_{i}^{b}\left(x,y\right)$ has an empty space in the left-side region. The right-side region with the intensity values is naturally lost as shown in Figure 4a. To fill the empty space, the proposed system reconstructs the image by attaching some parts of the neighboring frame as shown in Figure 4a,b. We then respectively generate the final reconstructed images ${\tilde{g}}_{i}^{b}\left(x,y\right)$ and ${\tilde{g}}_{i}^{a}\left(x,y\right)$ by attaching appropriate regions of the neighboring images onto $g_{i}^{b}\left(x,y\right)$ and $g_{i}^{a}\left(x,y\right)$ since the left-side facility of $g_{i}^{a}\left(x,y\right)$ is sometimes lost in the image acquisition process. Although some regions are duplicated, we can prevent from skipping the inspection of the regions. The lost region in $g_{i}^{b}\left(x,y\right)$ is used when reconstructing neighbor frames at the previous or next inspection stage.

### 3.3. Facility Detection Using Convolutional Neural Network

To automatically inspect railway facilities, we should find out their positions and classify types of them since each type has different risk management standard for a deformed area. A simple approach is to define absolute positions of all facilities in advance. However, it is inefficient since we should change the facility positions whenever the reference image set is replaced by new ones.

The proposed system detects facilities using deep convolutional neural network (CNN). Object detection based on deep learning was rapidly developed with various network models. Although recently proposed models have complicated structures with many layers for high accuracy, their detection speed tends to increase due to enormous number of parameters to be trained. We should also consider the detection accuracy using the dataset consisting of the grayscale images acquired from the line scan camera. Since images have only one channel, it is difficult to apply a segmentation-based detection method using color images. An advantage of the proposed system is that does not need a complex network model since the dataset used in the proposed system is simple and monotonous. Most facilities of all classes have similar shapes and sizes in the image set, so we do not need to design a complex detection network.

**Figure 5:**
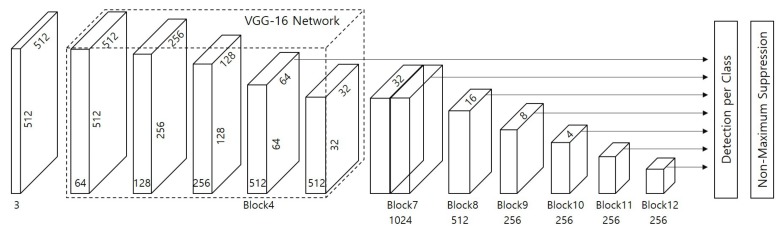
Network model of the original SSD with the input size of 512×512 [20].

To detect elements and facilities, the proposed system uses a deep neural network model that was modified from the original single-shot multibox detector (SSD) [20]. Figure 5 shows the SSD network model that takes an 512×512 image as input. The SSD falls into the category of the one-stage detector consisting of feed forward convolutional feature layers. Given an input image, it extracts feature maps using $c_{i}\times 3\times 3\times c_{i+1}$ convolution filters based on VGG model [21] as the baseline network at each layer, where $c_{i}$ represents the number of channels of the *i*-th layer. Objects are detected using multiple anchor boxes and softmax classifiers during the convolution process. Since the VGG has a down-scaled feature pyramid structure with a few layers, the SSD has the fast and accurate performance. However, the VGG needs a huge number of parameters for training even with simple and relatively shallow structure. Some improved network architectures derived from the SSD were proposed to improve the detection accuracy at the cost of lower speed due to the increasing computational complexity. Fortunately, the proposed system does not need to design more complex and deeper network architecture than the original SSD. Facilities of the same class in all images have similar sizes, shapes, and box ratios as shown in Figure 2. It allows easily detecting them by reducing the number of convolution layers or feature channels even if the input images captured by the line scan camera have a single channel.

**Figure 6:**
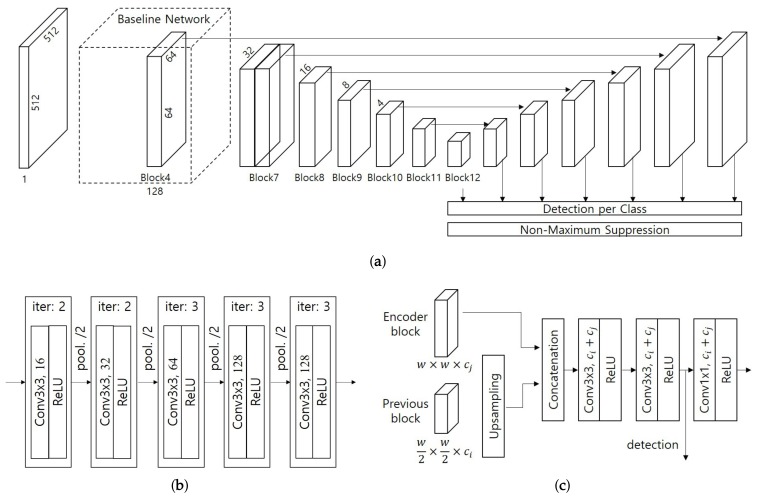
Network architecture of the proposed system: (**a**) overall network architecture; (**b**) baseline network using the VGG model; and (**c**) decoding block for object detection.

Figure 6a shows the network architecture of the improved SSD. The network starts the detection process using the grayscale input image of size 512×512×1. We used the VGG model as the baseline network, where the number of channels in each convolution layer is a quarter of the number of feature channels as shown in Figure 6b. Although it enables a quick detection performance, the accuracy decreases due to the reduced number of parameters. More specifically, the accuracy in detecting small objects rapidly decreases since the relatively shallow network loses features in a small object. To improve that drawback, we introduced additional blocks derived from the deconvolutional SSD proposed by Fu et al. [22]. As shown in Figure 6a, the network model has an auto-encoder containing upsampling layers of the same size as the corresponding blocks. Figure 6c shows the details of the decoding block. The feature map extracted in the previous block is concatenated with its corresponding block in the encoding model after passing through the upsampling layer. In the proposed network, the number of encoding feature maps is the half of the detecting block used in the original SSD. Followed by the encoder network, two convolution layers with rectified linear units are added to mix the concatenated feature channels. We use the result for object detection without a prediction module, which was used in the DSSD, to reduce the number of learning parameters, and then reduce the number of channels by half using the 1×1 convolution layer to repeat the decoding process. Consequently, the proposed network keeps the one stage and single-shot model using more deep layers and reduced number of parameters.

**Figure 7:**
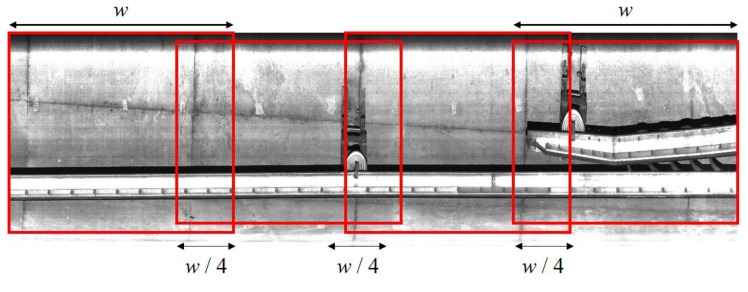
Split strategy of the proposed detection algorithm.

In the training process, we reduce the training images down to one quarter by splitting the input image with the width-to-height ratio of 8:1 into sub-images with the ratio of 1:1. We also use the same loss functions in the original version. In the detection process, the proposed system splits the reconstructed image to improve the detection accuracy. Figure 7 shows the detection strategy of the proposed system. When the images split, shapes of a facility are divided into two neighboring sub-images. The proposed system splits the image with overlapping between neighboring sub-images. Since the detection results are overlapped at the same facility, the proposed system combines the results by selecting their minimum and maximum coordinates when they overlap the area of 50 percentage. Next, the proposed system uses the phase correlation again to match and align the results of two images, ${\tilde{g}}_{i}^{b}\left(x,y\right)$ and ${\tilde{g}}_{i}^{a}\left(x,y\right)$. We obtain the center coordinates of comparing results of ${\tilde{g}}_{i}^{a}\left(x,y\right)$, and then find the most similar objects by selecting the position with the maximum similarity. When the detector finds the same object with a difference size in ${\tilde{g}}_{i}^{b}\left(x,y\right)$ and ${\tilde{g}}_{i}^{a}\left(x,y\right)$, we select the bigger bounding box. The proposed system sets the size of bounding box if it is detected in one of two images. Consequently, we obtain the detection results as pairs of bounding boxes detected respectively in ${\tilde{g}}_{i}^{b}\left(x,y\right)$ and ${\tilde{g}}_{i}^{a}\left(x,y\right)$.

### 3.4. Facility Inspection

#### 3.4.1. Preprocessing

**Figure 8:**
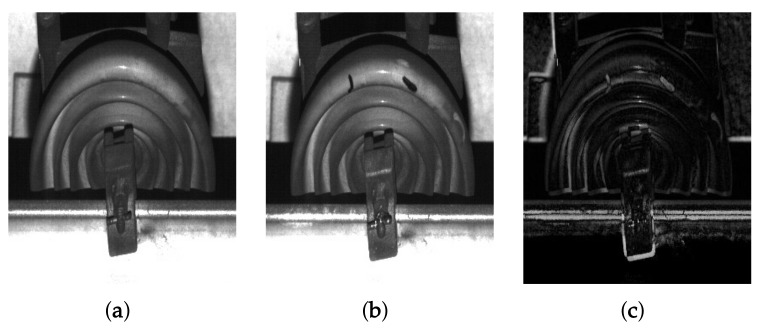
Subtraction result of a pair of facility images: (**a**,**b**) are facility images which we should compare, and (**c**) their subtraction result. (**a**) was captured in advance before (**b**).

The proposed system finds cracks in a facility by comparing a pair of images acquired at the same position. Although conventional methods can find thin cracks using a single image, they are not suitable for wide-area inspection. On the other hand, multiple image-based method, that is an image comparison approach, works well in thee wide-area inspection since it computes the difference of two comparing images by simple image subtraction. The results also include deformed areas of facilities for prevention of potential risk. However, multiple image-based inspection systems have some issues. Figure 8 shows a subtraction result of two images with a facility detected at the same position. The simple subtraction gives rise to an inaccurate result since the operator is dependent on intensity values of two comparing images. Although the proposed system performs image registration at the previous steps, the result has unexpected errors in the shape of facility because of train’s jittering and inconsistent velocity when comparing images are acquired at different times.

**Figure 9:**
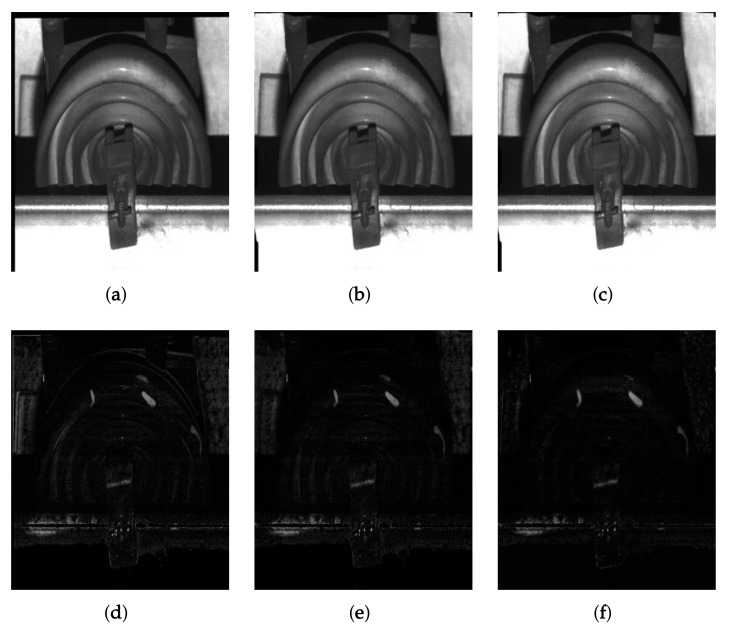
Result of each preprocessing step: (**a**) transformed image of ${\tilde{g}}_{i,j}^{b}\left(x,y\right)$ using feature matching and homography, (**b**) warped image of (**a**) using optical flow-based registration, (**c**) transferred result of (**b**) using histogram specification, and (**d**–**f**) subtraction results of (**a**–**c**) by the absolute difference operation, respectively.

To solve these problems, the proposed system first transforms the facility image of ${\tilde{g}}_{i}^{b}\left(x,y\right)$ similarly to the image of ${\tilde{g}}_{i}^{a}\left(x,y\right)$ using a feature matching approach. Given a pair of *j*-th facility images cropped using the bounding box, ${\tilde{g}}_{i,j}^{b}\left(x,y\right)$ without any defect and ${\tilde{g}}_{i,j}^{a}\left(x,y\right)$ with potential wears and cracks, we match the corresponding features between two comparing images using speeded-up robust feature (SURF) [23] and random sample consensus (RANSAC) algorithm [24]. The proposed system then transforms ${\tilde{g}}_{i,j}^{b}\left(x,y\right)$ by estimating the homography among the corresponding features of ${\tilde{g}}_{i,j}^{b}\left(x,y\right)$ and ${\tilde{g}}_{i,j}^{a}\left(x,y\right)$ as shown in Figure 9a. Figure 9d shows the subtraction result using feature matching and geometric transformation. The result is better than the simple subtraction as shown in Figure 8c.

Next, the proposed system performs a non-rigid registration using motion field. Unlike the homography-based approach that globally transforms the image by keeping the rectangular shape, the non-rigid registration is robust to local transformation of regions because each motion represents a displacement with orientation in the image space. To locally register the transformed image, we obtain the motion field using the optical flow estimation method that estimates dense motion vectors in all pixels using expansion of a quadratic polynomial [25]. The proposed system then obtains the registered image by warping the transformed image using the motion field as shown in Figure 9b. Figure 9e shows the subtraction result between the registered image and the potentially deformed image. The shapes of two images are almost the same with less errors than the result of Figure 9d, but some errors still remain due to the difference of the brightness.

To match the brightness level of ${\tilde{g}}_{i,j}^{a}\left(x,y\right)$ into the warped image, the proposed system performs histogram specification. We match the intensity values of two images as

(2) $${\bar{g}}_{i,j}^{b}\left(x,y\right)=\left\{\begin{array}{cc} T_{a}^{-1}\left[T_{b}\left({\tilde{g}}_{i,j}^{b}\left(x,y\right)\right)\right], & |T_{a}^{-1}\left[T_{b}\left({\tilde{g}}_{i,j}^{b}\left(x,y\right)\right)\right]-{\tilde{g}}_{i,j}^{a}\left(x,y\right)|<|{\tilde{g}}_{i,j}^{b}\left(x,y\right)-{\tilde{g}}_{i,j}^{a}\left(x,y\right)|\\ {\tilde{g}}_{i,j}^{b}\left(x,y\right), & \mathrm{elsewise} \end{array}\right.,$$

where ${\bar{g}}_{i,j}^{b}\left(x,y\right)$ represents the intensity-matched version of ${\tilde{g}}_{i,j}^{b}\left(x,y\right)$, and $T_{b}^{-1}\left(\cdot \right)$ and $T_{a}^{-1}\left(\cdot \right)$ are the cumulative density functions of ${\tilde{g}}_{i,j}^{b}\left(x,y\right)$ and ${\tilde{g}}_{i,j}^{a}\left(x,y\right)$, respectively. |·| indicates the absolute function. Since the histogram specification is considered to be a global intensity transfer function, some regions in the reference image may become saturated. For that reason, the proposed system decreases the subtraction error by adding the condition as shown in Figure 9f, where the histogram matching method selects the original intensity if the absolute difference with ${\tilde{g}}_{i,j}^{a}\left(x,y\right)$ is lower than the difference between the transformed result of ${\tilde{g}}_{i,j}^{b}\left(x,y\right)$ and ${\tilde{g}}_{i,j}^{a}\left(x,y\right)$.

#### 3.4.2. Detection of Candidate Defects in the Facility

When the shapes between two comparing images are well-matched with the low difference of intensity values, the main issue of the proposed system is to detect a deformed area with an existence of noise. It is difficult to remove the noise in an image since some small cracks may be removed together with the noise. For that reason, the proposed system extracts the candidate defect regions by excluding the noise as much as possible. We first obtain a weight for modification of the subtraction result between ${\bar{g}}_{i,j}^{b}\left(x,y\right)$ and ${\tilde{g}}_{i,j}^{a}\left(x,y\right)$ to reduce the common high-frequency components as

(3) $$e_{i,j}\left(x,y\right)=\left[1-\{\alpha \cdot e_{i,j}^{b}\left(x,y\right)+\left(1-\alpha \right)\cdot e_{i,j}^{a}\left(x,y\right)\}\right],$$

where $e_{i,j}\left(x,y\right)$ represents the weight for the edge area, $e_{i,j}^{b}\left(x,y\right)$ and $e_{i,j}^{a}\left(x,y\right)$ respectively high-frequency magnitudes of ${\bar{g}}_{i,j}^{b}\left(x,y\right)$ and ${\tilde{g}}_{i,j}^{a}\left(x,y\right)$ using Prewitt operator, and α weight for the magnitudes.

**Figure 10:**
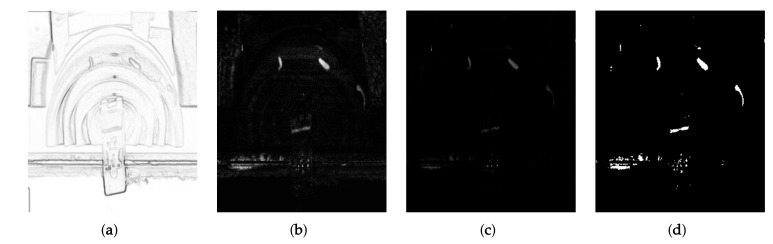
(**a**) Weight for reduction of the common high-frequency components; (**b**) multiplication result of (**a**) with the subtraction result; (**c**) Weber contrast of ${\bar{g}}_{i,j}^{b}\left(x,y\right)$ and ${\tilde{g}}_{i,j}^{a}\left(x,y\right)$; and (**d**) defect candidate image. Note that (**b**–**c**) were expressed by normalizing the original result.

Next, the proposed system obtains candidate of deformed regions. Although the subtraction result is improved by multiplying $e_{i,j}\left(x,y\right)$, some errors still remain due to the noise and small registration error as shown in Figure 10b. To solve the problem, we use another weight using the Weber-Fechner’s law, which relates a perceptual stimulus change of the human vision to the initial stimulus level [26].

When we assume that the physical stimulus is equivalent to the intensity value in an image, the Weber contrast $w_{i,j}\left(x,y\right)$ is defined as

(4) $$w_{i,j}\left(x,y\right)=\frac{\Delta I}{I}=\frac{{\bar{g}}_{i,j}^{b}\left(x,y\right)-{\tilde{g}}_{i,j}^{a}\left(x,y\right)}{{\bar{g}}_{i,j}^{b}\left(x,y\right)},$$

where *I* and ΔI represent the original stimulus and its change, respectively. Since the proposed system aims to detect wears and cracks by comparing the non-defective image ${\bar{g}}_{i,j}^{b}\left(x,y\right)$ and potentially defective image ${\tilde{g}}_{i,j}^{a}\left(x,y\right)$, we can define the fractional relation of the stimulus change for the initial stimulus as Equation (4). Figure 10c shows the Weber contrast result. Despite the unexpected results at the common dark area of ${\bar{g}}_{i,j}^{b}\left(x,y\right)$ and ${\tilde{g}}_{i,j}^{a}\left(x,y\right)$, some deformed regions are more remarkable than others with the background and the noise because the Weber contrast finds the small intensity differences and more clearly expresses them as the intensity level [27].

We finally obtain the reliable defect candidates by multiplying two weights with the subtraction results as

(5) $$d_{i,j}\left(x,y\right)=\left\{\begin{array}{cc} 1, & e_{i,j}\left(x,y\right)\cdot w_{i,j}\left(x,y\right)\cdot \left|{\bar{g}}_{i,j}^{b}\left(x,y\right)-{\tilde{g}}_{i,j}^{a}\left(x,y\right)\right|>T\\ 0, & \mathrm{elsewise} \end{array}\right.,$$

where $d_{i,j}\left(x,y\right)$ represents the results with the candidate defect regions and *T* the thresholding value. As shown in Figure 10d, we can obtain the candidate detection result reduced errors with the noise from the subtraction result of Figure 9f.

#### 3.4.3. Defect Region Decision Using Morphological Processing

Given a candidate of the defect image, the proposed system detects the deformed area using morphological processing. We first remove tiny regions obtained by the noise as

(6) $${\hat{d}}_{i,j}\left(x,y\right)=d_{i,j}\left(x,y\right)-\left[d_{i,j}\left(x,y\right)⋂\left\{d_{i,j}^{\eta }\left(x,y\right)⊕s_{2p+1}\right\}\right],$$

where ${\hat{d}}_{i,j}\left(x,y\right)$ represents the candidate image without the small regions including the noise, $s_{2p+1}$ structuring element with the size of (2p+1)×(2p+1) satisfying p≥0, and ⊕ indicates the morphological dilation operator. $d_{i,j}^{\eta }\left(x,y\right)$ is the binary image containing the center point of the tiny regions and is defined as

(7) $$d_{i,j}^{\eta }\left(x,y\right)=\left\{\begin{array}{cc} 1, & \sum_{u=-(p+1)}^{p+1}\sum_{v=-(p+1)}^{p+1}d_{i,j}\left(x+u,y+v\right)-\sum_{u=-p}^{p}\sum_{v=-p}^{p}d_{i,j}\left(x+u,y+v\right)=0\\ 0, & \mathrm{elsewise} \end{array}\right..$$

**Figure 11:**
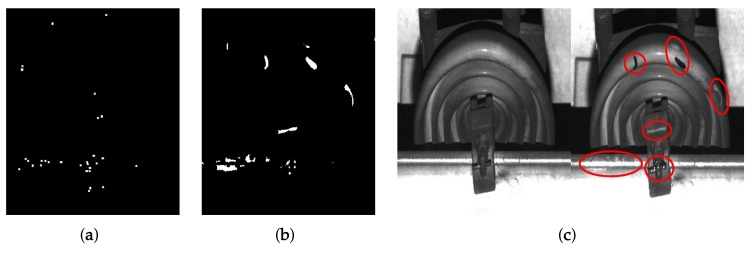
Defect detection result: (**a**) noisy image; (**b**) detected defect regions using the proposed system; and (**c**) a pair of image with the deformed regions.

Figure 11a shows the noise image obtained using Equation (7) and the structuring element with p=2. We can more efficiently remove the tiny regions than morphological erosion since the operation removes the thin area with the noise.

Consequently, the proposed system obtains the final result with the defect region, $r_{i,j}\left(x,y\right)$, by performing morphological closing operator as

(8) $$r_{i,j}\left(x,y\right)=\left\{{\hat{d}}_{i,j}\left(x,y\right)⊕s_{q}\right\}⊖s_{q},$$

where *q* denotes the size of structuring element and ⊖ indicates an erosion operator. Figure 11b shows the result of defect detection. The proposed system detects defect regions where most of the noise is removed in the candidate image when comparing the deformed regions as shown in red circles of Figure 11c.

## 4. Experimental Results

In this section, we demonstrate the performance of the proposed inspection system. We performed experiments under the environment of 2.2 GHz CPU, 64 GB RAM, and a Nvidia Geforce GTX 1080 Ti GPU. We implemented overall functions of the proposed system using C++ language with OpenCV library, and the detection function using Python tool with Tensorflow library. For experiments, we acquired a pair of image sets using a line scan camera in a tunnel of subway line 9 in Seoul, South Korea.

**Figure 12:**
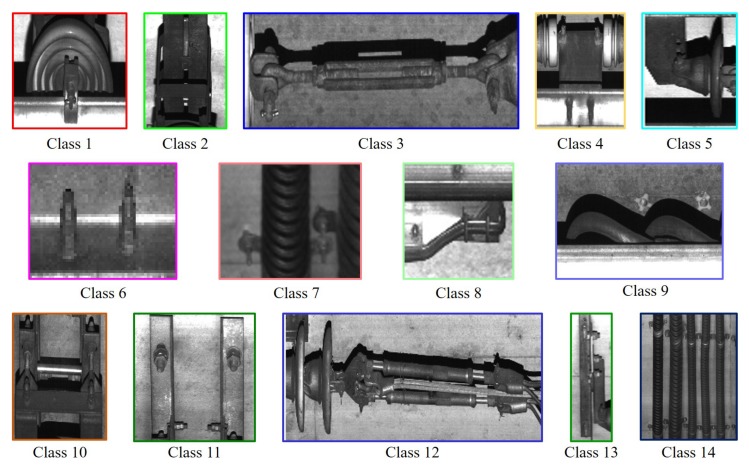
Classes forfacility detection used in the experiment.

In the first experiment, we tested the improved SSD to verify the performance. We trained all comparing networks with the proposed network 3 times using different learning rates, 0.01 for 200 K, 0.001 and 0.0001 for 50 K, sequentially. Some common functions are used, the loss function used in the original SSD, Adam optimizer built in the Tensorflow, the batch size of 8, and the weight decay of 0.0005. We trained the proposed network using 14,000 non-defect images with each size of 1:1 ratio to detect facilities of 15 classes with the background as shown in Figure 12. To evaluate the proposed network, we used a database consisting of the similar number of images with some potential defects.

We tested the performance of the proposed network by comparing with other networks designed using the original SSD (SD+VG). Table 1 shows layer configurations of five comparing networks.

SD+res50 is designed using the residual network [28] composed of 50 layers with bottleneck structure. We trained the SD+res50 using the same number of feature channels of the SD+VG model. SD+VG/2 has the same structure with the original model, but we reduced all feature map sizes as a half of each layer of the SD+VG. DSD+VG/2 and DSD+VG/4 are designed using the proposed network. The baseline model of two networks is same with SD+VG/2, but DSD+VG/4 has each half of the number of feature channels. All networks were trained using the same training condition with the proposed network.

**Figure 13:**
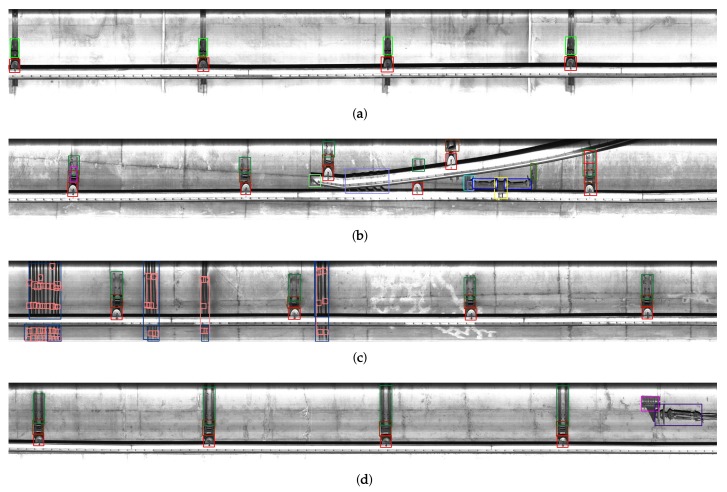
Detection results using the proposed network: (**a**–**d**) are results of the 1st, 16th, 20th, and 82th frame, respectively. Color of bounding boxes represents each class type as shown in Figure 12.

Table 2 and Table 3 show detection results in the sense of mean of average precision (mAP) and computational time per image, respectively. We set thresholding values, detection score of 0.7 and overlapping area of bounding box of 0.3 for non-maximum suppression. The performance of SD+res50 is lower than others designed using the VGG baseline network model. SD+VG/2 has the similar mAP, but the computational time is faster than SD+VG. It reminds that many parameters and layers do not decide the performance of the network. In addition, this result verifies that light network model can have good performance when simple dataset is trained. The proposed network-based models, DSD+VG/2 and DSD+VG/4, have better mAPs and computational times than other networks. Similar to the result of the comparison with SD+VG and SD+VG/2, DSD+VG/4 is a bit faster than DSD+VG/2 even if the similar mAPs of two networks. As shown in Figure 13, the proposed network can accurately detect facilities.

Next, we tested the second experiment to measure the performance of the defect detection. In the experiments, we acquired two image sets using the line scan camera at the same section but different times. Since the database are acquired using the camera installed on the vehicle providing transportation service, we could not manipulate facilities for safety reasons. Alternatively, we simulated some defects at the image set captured after another set using a commercial image painting tool by considering possible defects such as insulator cracks and fastener loosing. We inserted 337 defects in 50 images with each ground truth (GT), and compared to their corresponding non-defect images using the proposed system. We set α=0.5 in (3) used for the edge weight and T=0.12 in (5) used for detection of candidate defect region. For quantitative evaluation, we measured the precision, recall, and intersection of union (IoU) as

(9) $$\mathrm{Precision}=\frac{TP}{TP+FP},$$

(10) $$\mathrm{Recall}=\frac{TP}{TP+FN},$$

(11) $$\mathrm{IoU}=\frac{TP}{TP_{GT}},$$

where TP, $TP_{GT}$, FP and FN represent the area of true positive, GT of true positive, false positive, and false negative, respectively. In addition, we measured a rate, defined as *hit rate*, using the number of detected results for the number of simulated defects as

(12) $$\mathrm{Hit}\;\mathrm{rate}=\frac{N^{TP}}{N^{GT}},$$

where $N^{TP}$ and $N^{GT}$ represent the number of detected defects and simulated defects, respectively. $N^{TP}$ is accumulated when the area of a defected defect per a simulated defect is bigger than 0.1. For the hit rate, we evaluate the accuracy of detecting significantly deformed regions for facility maintenance.

**Figure 14:**
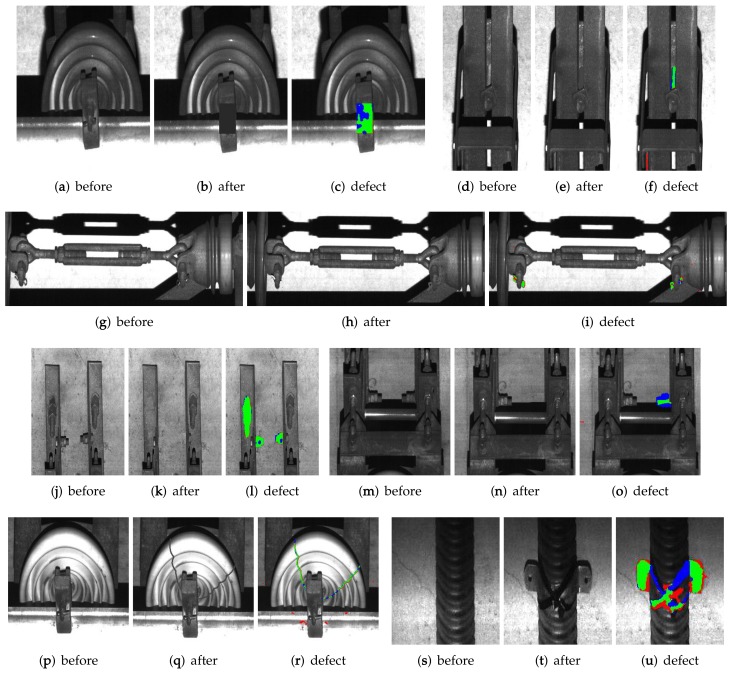
Results of deformed regions using the proposed system. Green, blue, and red color represent true positive, false negative, and false positive, respectively.

Table 4 and Figure 14 show the quantitative and qualitative evaluation result of the proposed system. In the experiment, we obtain relatively low values of precision, recall, and IoU. Since the proposed system compares a pair of railway tunnel images by relying on their intensity values and the constant thresholding value, inaccurate results are sometimes obtained if the brightness difference of images are low. Nevertheless, the proposed system can accurately detect noticeable defect regions as shown in the hit rate of Table 4.

Table 5 shows the computational time of the proposed system represented as second per an image. We implemented most modules except the detection network using C++ language, and loaded the detection module implemented using Python in the proposed system. For improvement of the inspection speed, we applied the OpenMP library for parallel processing in some methods. As shown in Table 5, the proposed system can quickly provide the inspection result for facility maintenance.

## 5. Conclusions

This paper presented a novel, deep learning-based railway inspection system for facility maintenance using image analysis in the urban railway field. Unlike conventional methods and systems, the proposed system finds wears and cracks by comparing a pair of images of the same location at different times. Line scan camera can overcome drawbacks of area-based camera that has the narrow FoV and low image acquisition speed. The proposed system inspects facilities using deep learning-based object detection. More specifically, an improved single shot detector was proposed to find and classify facilities with better performance than the original SSD using the dataset acquired in the subway tunnel environment, although networks used in the experiment for detection have high accuracies since facilities of the same class have similar shapes and sizes. The proposed system finds facility defects using image comparison approach based on the absolute difference and the Weber’s law. Image comparison based on the difference measurement is more efficient than single image-based analysis because we can simply find the difference of a pair of images including respectively normal and abnormal facilities. The Weber contrast is also suitable to compare a pair of images since its nominator and denominator represent the image difference as relative stimulus change and the intensity of the reference image as initial stimulus, respectively.

The proposed system can provide various benefits in managing railway infrastructure. Specifically, we can monitor facilities at all times and maintain any fault and defect to prevent accidents if the proposed system is equipped in commercial vehicles for transportation. It can also reduce cost and time for inspection. Consequently, the proposed system can provide an automatic inspection and maintenance of urban railway infrastructure.

## Figures and Tables

**Table 1:** Layer specification of each network model.

	SD+VG		SD+res50		SD+VG/2		DSD+VG/2		DSD+VG/4	
	iter.	layer	iter.	layer	iter.	layer	iter.	layer	iter.	layer
**Block 1**	2	3×3, 64	1	7×7, 64	2	3×3, 32	2	3×3, 32	2	3×3, 16
**Block 2**	2	3×3, 128	1	3×3, 128	2	3×3, 64	2	3×3, 64	2	3×3, 32
				1×1, 32						
			3	3×3, 32						
				1×1, 128						
**Block 3**	3	3×3, 256	1	3×3, 256	3	3×3, 128	3	3×3, 128	3	3×3, 256
				1×1, 64						
			4	3×3, 64						
				1×1, 256						
**Block 4**	3	3×3, 512	1	3×3, 512	3	3×3, 256	3	3×3, 256	3	3×3, 128
				1×1, 128						
			6	3×3, 128						
				1×1, 512						
**Block 5**	3	3×3, 512	1	3×3, 512	3	3×3, 256	3	3×3, 256	3	3×3, 128
				1×1, 128						
			4	3×3, 128						
				1×1, 512						
**Block 6**	1	3×3, 1024	1	3×3, 1024	1	3×3, 256	1	3×3, 256	1	3×3, 128
**Block 7**	1	1×1, 1024	1	1×1, 1024	1	1×1, 512	1	1×1, 512	1	1×1, 256
**Block 8**	1	1×1, 256	1	1×1, 256	1	1×1, 128	1	1×1, 128	1	1×1, 64
	1	3×3, 512	1	3×3, 512	1	3×3, 256	1	3×3, 256	1	3×3, 128
**Block 9-12**	1	1×1, 128	1	1×1, 128	1	1×1, 64	1	1×1, 64	1	1×1, 32
	1	3×3, 256	1	3×3, 256	1	3×3, 128	1	3×3, 128	1	3×3, 64
**Block 13-14**							2	3×3, 128	2	3×3, 128
								1×1, 64		3×3, 64
**Block 15**							2	3×3, 128	2	3×3, 128
								1×1, 128		3×3, 128
**Block 16**							2	3×3, 256	2	3×3, 256
								1×1, 256		3×3, 256
**Block 17**							2	3×3, 512	2	3×3, 512
								1×1, 128		3×3, 128
**Block 18**							2	3×3, 256	2	3×3, 256

**Table 2:** Mean of average precision (mAP) of facility detection (%). Red and blue numbers are the best and second probabilities in each column, respectively.

Model	1	2	3	4	5	6	7	8	9	10	11	12	13	14	Total
**SD+VG**	99.8	99.6	100	94.8	**89.1**	96.2	**96.2**	100	**99.8**	99.5	98.7	100	**82.4**	**99.9**	96.8
**SD+VG/2**	99.8	99.6	100	92.6	86.1	96.5	94.0	100	99.5	**99.5**	97.9	100	**86.0**	98.9	96.5
**SD+res50**	99.2	**99.8**	99.4	97.2	84.2	90.0	72.1	99.2	94.1	98.8	98.6	100	69.6	98.4	92.9
**DSD+VG/2**	**99.9**	**99.8**	100	**98.4**	87.9	**97.9**	**96.0**	100	**99.5**	**99.8**	**99.7**	100	79.0	99.4	**96.9**
**DSD+VG/4**	**99.9**	99.4	100	**99.7**	**91.1**	**98.0**	91.7	100	99.4	99.4	**99.4**	100	79.5	**99.6**	**96.9**

**Table 3:** Computational time of network models (ms).

	SD+VG	SD+VG/2	SD+res50	DSD+VG/2	DSD+VG/4
**Time (ms)**	106	87	107	105	100

**Table 4:** Quantitative evaluation for the defect detection module.

	Precision	Recall	IoU	Hit Rate
**Value**	0.5420	0.7243	0.4224	0.9007

**Table 5:** Computational time of the proposed system (sec/image).

	Mean	Min	Max
**Image Loading**	1.090	0.120	1.249
**Reconstruction**	0.511	0.400	0.584
**Facility Detection**	0.450	0.360	0.535
**Facility Inspection**	0.822	0.366	3.952
**Total**	3.260	1.877	6.866
